# Copy Number Studies in Noisy Samples

**DOI:** 10.3390/microarrays2040284

**Published:** 2013-11-06

**Authors:** Philip Ginsbach, Bowang Chen, Yanxiang Jiang, Stefan T. Engelter, Caspar Grond-Ginsbach

**Affiliations:** 1 Neurology Department, University of Heidelberg, INF 400, Heidelberg D69120, Germany; E-Mails: Philip.Ginsbach@t-online.de (P.G.); jiangyanxiang@googlemail.com (Y.J.); 2 Division of Molecular Genetic Epidemiology, German Cancer Research Center, INF 280, Heidelberg D69120, Germany; E-Mail: c.bowang@dkfz-heidelberg.de; 3 Stroke Unit and Department of Neurology, University Hospital Basel, Petersgraben 4, Basel CH4031, Switzerland; E-Mail: Stefan.Engelter@usb.ch

**Keywords:** copy number variation (CNV), variance, wave noise, per-SNP noise, noise-free-cnv software, noise reduction, validation of CNV findings

## Abstract

System noise was analyzed in 77 Affymetrix 6.0 samples from a previous clinical study of copy number variation (CNV). Twenty-three samples were classified as eligible for CNV detection, 29 samples as ineligible and 25 were classified as being of intermediate quality. New software (“noise-free-cnv”) was developed to visualize the data and reduce system noise. Fresh DNA preparations were more likely to yield eligible samples (*p* < 0.001). Eligible samples had higher rates of successfully genotyped SNPs (*p* < 0.001) and lower variance of signal intensities (*p* < 0.001), yielded fewer CNV findings after Birdview analysis (*p* < 0.001), and showed a tendency to yield fewer PennCNV calls (*p* = 0.053). The noise-free-cnv software visualized trend patterns of noise in the signal intensities across the ordered SNPs, including a wave pattern of noise, being co-linear with the banding pattern of metaphase chromosomes, as well as system deviations of individual probe sets (per-SNP noise). Wave noise and per-SNP noise occurred independently and could be separately removed from the samples. We recommend a two-step procedure of CNV validation, including noise reduction and visual inspection of all CNV calls, prior to molecular validation of a selected number of putative CNVs.

## 1. Introduction

Genomic copy number variation (CNV) was associated with a variety of clinical phenotypes [1,2,3,4,5,6]. Hence, the study of CNV is of diagnostic importance. CNV identification from high-density SNP-microarrays may be unreliable, particularly in noisy data [7,8,9]. Therefore, extensive validation of CNV findings is needed. Since CNV detection software may identify hundreds of putative CNVs in each sample and since validation of CNV findings by qPCR, or by other molecular methods, is laborious, we searched for simple strategies to evaluate large numbers of CNV findings.

Rigorous studies revealed that several components of system error occur in copy number data [10,11,12,13]. Here we focus on two major types of noise and present the noise-free-cnv software package for the visualization of copy number data and for the reduction of noise. This software enables large-scale inspection of CNV findings (produced by PennCNV [14], Birdview [15,16], or other specialized software packages). For illustration, we used 77 microarrays from a previous study of patients with cervical artery dissection from Switzerland and Southern Germany (age: 42.5 ± 9.8 years; 31 (40.3%) women) [17]. DNA was isolated from peripheral blood samples (no DNA from lymphoblastoid cell lines was used). DNA extraction, array hybridization, and array scanning were performed according to the manufacturer’s instructions [17]. The LRR and BAF values were obtained from the CEL files with the Affymetrix Power Tools software (APT). The quantile normalization was done in APT. The LRR and BAF can be then imported to PennCNV, to other CNV detections software packages (QuantiSNP, MAD), or to noise-free-cnv.

The Affymetrix 6.0 microarrays used for CNV detection contain a total of 906,600 single nucleotide polymorphisms (SNPs) and 946,000 non-polymorphic copy number probes (CNPs) covering all human chromosomes. In the present article, the notion of SNP is used for all analyzed probe sets (SNPs as well as CNPs).

## 2. Noise Components

**Figure 1:**
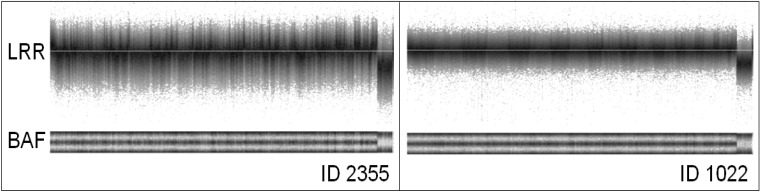
Signal strength (LRR) and B-allele frequency (BAF) of samples from two male patients (ID 2355 and ID 1022). SNPs were visualized in increasing position along the chromosomes. LRR values of patient ID 2355 have larger variance and show pronounced wave noise.

**Figure 2:**
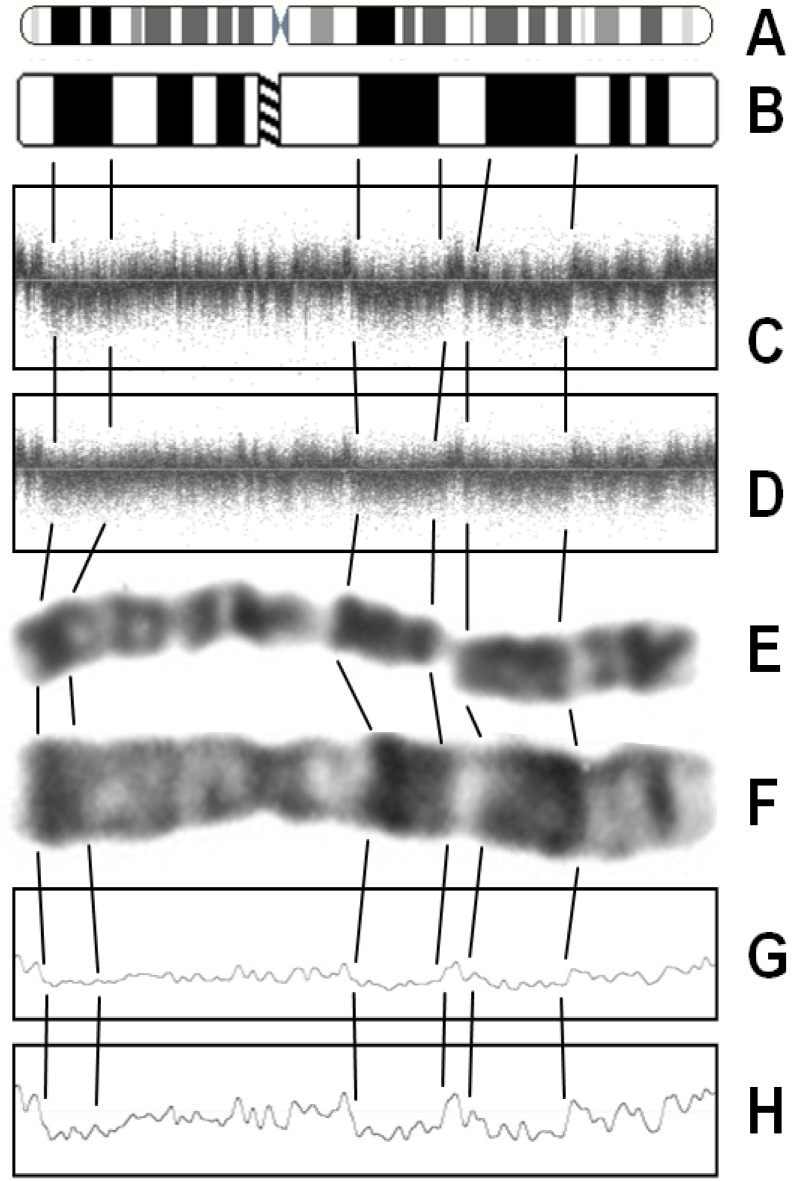
Wave noise. Ideograms of pro-metaphase (**A**) and metaphase (**B**) chromosome 7 were compared with signal intensities of SNPs of chromosome 7 of two patients (**C**,**D**) and with a human prometaphase (**E**) and metaphase (**F**) chromosome 7. Signal intensities shown in C and D were smoothed (noise-free-cnv software, function “blur” across 1,000 probe sets) to visualize genomic waves (**G**,**H**).

Figure 1 shows two samples (visualized by noise-free-cnv), displaying signal intensity (LRR—upper panel) and B-allele frequency (BAF—lower panel) of all SNPs ordered along the chromosomes. The Log R Ratio (LRR) is a normalized measure of the total signal intensity for two alleles of the SNP. The B-Allele Frequency (BAF) is a normalized measure of the allelic intensity ratio of two alleles [18]. Signal intensities in sample ID 2355 show larger variance than in ID 1022. Moreover, a prominent pattern of waves is apparent in sample ID 2355. In many samples, we observed similar wave patterns. The noise-free-cnv software identified waves using a Gaussian filter with a large standard deviation, for instance comprising 1,000 SNPs. This filter “blurs” the values as shown in Figure 2(G,H). We called the resulting wave data the *wave component* of the LRR values. The variance of the blurred LRR values is a measure for the prominence of waves, the *wave variance.*

This wave pattern was compared with the banding pattern of metaphase chromosomes (Figure 2). Human metaphase chromosomes were stained with the Giemsa-trypsine procedure, which induces a banding pattern. AT-rich regions are more frequent in Giemsa-dark bands than in Giemsa-light bands [19,20]. In our study samples, Giemsa-dark bands corresponded to genomic regions with reduced probe set signals. This pattern of noise was described by others as “genomic waves” or “CG-waves” [10,11,12,13]. The co-linearity of genomic waves with Giemsa bands illustrates that genomic waves follow a similar pattern in all samples.

**Figure 3:**
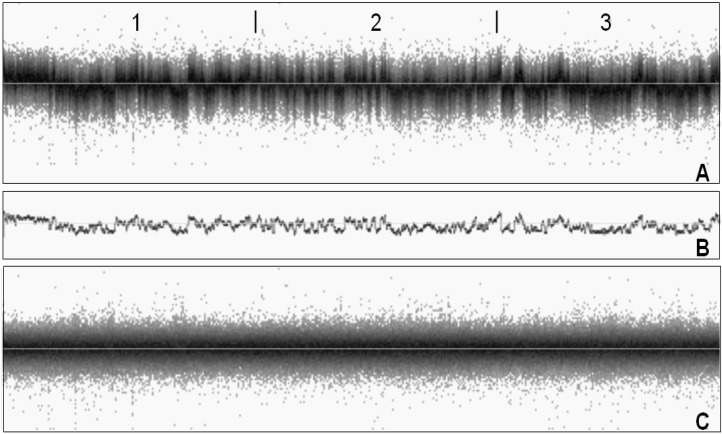
Noise components. LRR values of a noisy sample (**A**), split up in wave component (**B**) and per-SNP component (**C**). All SNPs of chromosomes 1–3 were shown (chromosomes indicated on top of panel A).

After subtraction of the wave component, the resulting LRR values follow an approximately normal distribution around zero. We called the resulting values *per-SNP component* and their variance the *per-SNP variance.* The decomposition of system noise in wave component and per-SNP component is shown for one sample in Figure 3. Wave variance and per-SNP variance components were calculated for all samples in Table A1.

**Figure 4:**
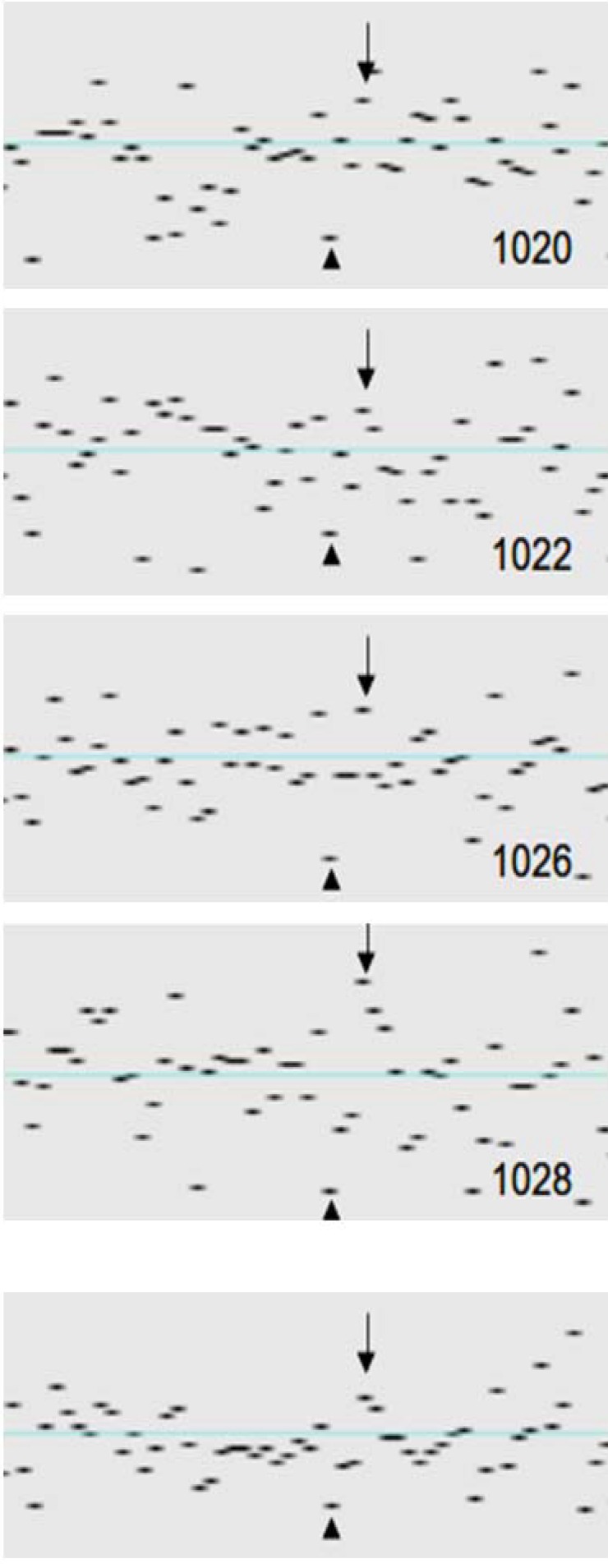
per-SNP system noise. Signal intensities in genomic region 2: 189766706–189891527 shown for four patients (ID 1020; ID 1022; ID1026; ID 1028). The lower panel shows the per-SNP median profile (median signal intensities) of all samples (*n* = 77). Arrows and arrowheads indicate SNPs with LRR values far above and below the mean.

The system deviations of individual SNP signal intensities are strongly correlated across samples (Figure 4). To quantify the correlation of the noise (variance) components between different samples, we computed two additional data series: for each SNP the median through all 77 per-SNP components was computed and saved as the *per-SNP profile*. For the *wave profile* the same procedure was applied to the wave components. We then computed, for each sample, the correlation between the wave profile and the (individual) wave component as well as the correlation between the per-SNP profile and the (individual) per-SNP component. Details of the algorithm are described in Appendix. The high correlations found in our 77 samples confirmed that wave noise and per-SNP noise are *system* noise, *i.e.*, follow highly non-random patterns. On average, the correlation was 0.843 for the wave component and 0.568 for the per-SNP component.

## 3. Factors Associated with Quality of Copy Number Data

The resolution of a classical chromosome study depends on the quality of the chromosomes and is expressed as the total number of visible cytogenetic bands (400 bands: low to moderate quality; 850 bands: excellent quality). According to our knowledge, no comparable quality metric for molecular karyotyping exists. Quality control in most copy number studies consists of rejecting samples with outlier numbers of CNV findings. A quality metric for the resolution of a CNV study (relating the size of a CNV and the likelihood of its detection) has not yet been defined.

**Figure 5:**
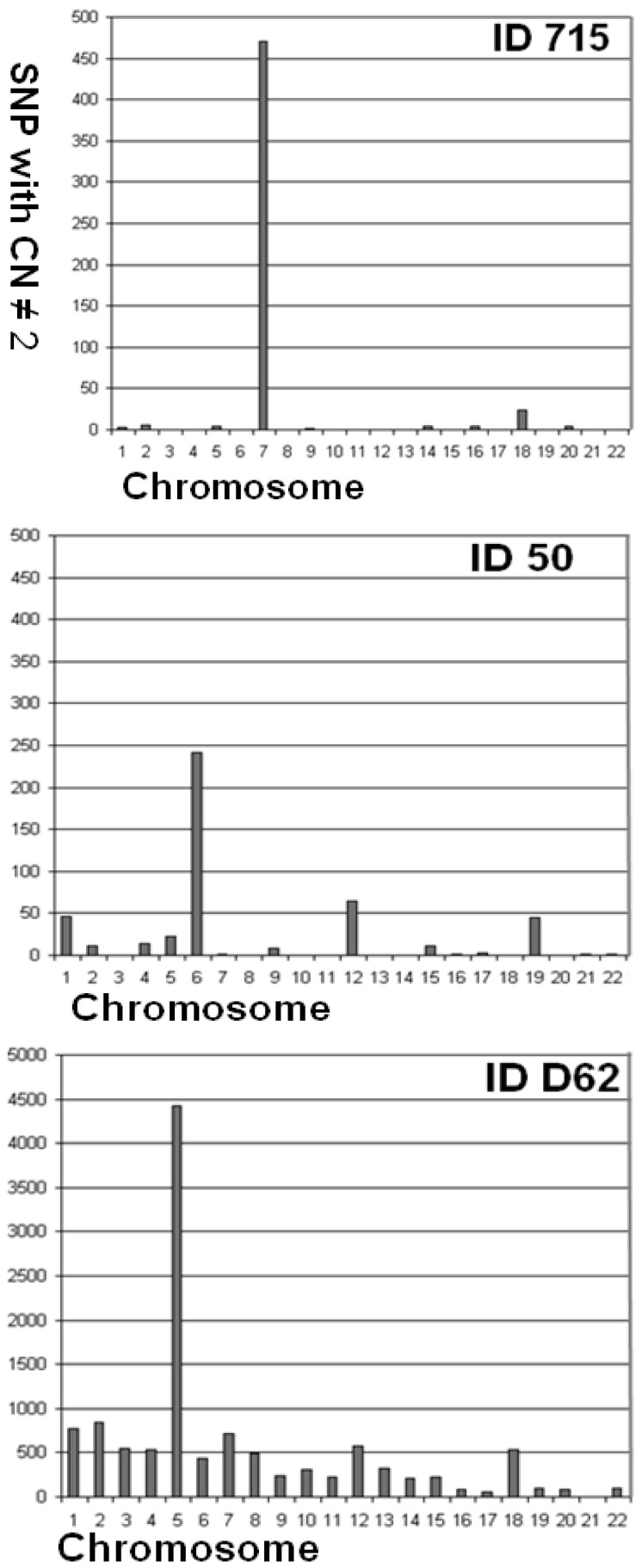
Quality of copy number samples. Number of SNPs with CN ≠ 2 per chromosome were scored. Sample ID 715 is eligible for CNV studies (most chromosomes without SNPs with CN ≠ 2). Accumulation of aberrant SNPs in chromosome 7 and 18 indicates presence of rare CNVs. Sample ID 50 is of intermediate quality. Sample ID 062 was classified as ineligible for CNV studies (>100 SNPs with CN ≠ 2 in most chromosomes).

In the current study we propose a preliminary quality metric based on the median number of SNPs per chromosome with copy number state (CN) ≠ 2 (numbers/chromosome for all cases are shown in Table A1). Copy Number state of each SNP was determined by the Affymetrix Power Tools software package (APT). SNPs located in common CNVs were excluded from this analysis. To identify SNPs located in common CNVs, we analyzed 403 control samples without visible waves and with highest genotype call rates selected from a large German population (PopGen [21]), as described before [17]. The median number of SNPs with CN ≠ 2 per chromosome was considered as a preliminary quality metric. The quality of a sample was related to the chromosomal background of SNPs with abnormal copy number (Figure 5). We defined deliberate quality categories: samples were classified as *eligible*, if the median number of SNPs per chromosome with CN ≠ 2 was zero, those with >100 SNPs with CN ≠ 2 were classified as *ineligible*.

Samples were classified according to the defined quality categories in Table 1. The use of freshly prepared DNA (compared to DNA samples that were used since years and had been thawed and frozen repeatedly) was a significant determinant of eligible samples (*p* < 0.001). Samples with high call rate (rate of successfully genotyped SNPs) were more likely to be suitable for copy number studies than those with lower call rates (*p* < 0.001). Low levels of wave variance as well as per-SNP variance were associated with eligibility for CNV analysis (*p* < 0.001). Eligibility for CNV studies was not significantly associated with the median number of calls by PennCNV (*p* = 0.053). However, eligible samples had between 63 and 165 calls, while the range of calls was much broader in ineligible samples. Birdview yielded significantly more calls in ineligible samples (*p* < 0.001). The proportion of putative false positive Birdview calls increased with decreasing confidence rates: The number of CNV findings with confidence below 2.5 was most strongly elevated.

**Table 1:** Characteristics of 77 analyzed samples, classified according to eligibility for copy number variation (CNV) analysis. Numbers indicate mean values and range (lowest–highest value). Mean values were compared between groups with the Chi-2 test or the Kruskal-Wallis test.

	Ineligible	Intermediate	Eligible	Chi-2/ kruskal-wallis
	(n = 29)	(n = 25)	(n = 23)	*p*
Fresh DNA preparation	0 (0.0 %)	6 (20.7 %)	14 (60.9 %)	<0.001
Genotyping call rate	94.7 [80.9–97.3]	96.6 [94.8–98.3]	97.7 [96.6–98.5]	<0.001
Autosomal variance	0.2291 [0.115–0.706]	0.1343 [0.068–0.208]	0.0870 [0.062–0.114]	<0.001
wave noise	0.0109 [0.002–0.058]	0.0034 [0.001–0.017]	0.0015 [0.001–0.013]	<0.001
per–SNP noise	0.2259 [0.082–0.696]	0.1281 [0.067–0.204]	0.0811 [0.060–0.164]	<0.001
PennCNV, No. of calls	238 [14–1821)	103 [34–1024]	98 [63–165]	0.053
PennCNV, % of deletions	18.6 [1.3–81.3]	27.4 [0.7–65.9]	40.0 [10.3–54.8]	0.164
Birdview No. of calls	527 [163–8,203]	225 [154–1,339]	208 [163–348]	<0.001
Birdview (cf > 10)	15 [2–717]	12 [5–33]	14 [4–20]	0.048
Birdview (cf = 10)	89 [76–145]	92 [74–105]	94 [77–102]	0.209
Birdview (cf 2.5–10)	93 [14–3344]	19 [10–361]	21 [11–45]	<0.001
Birdview (cf < 2.5)	370 [52–5665]	106 [35–857]	85 [42–194]	<0.001

**Figure 6:**
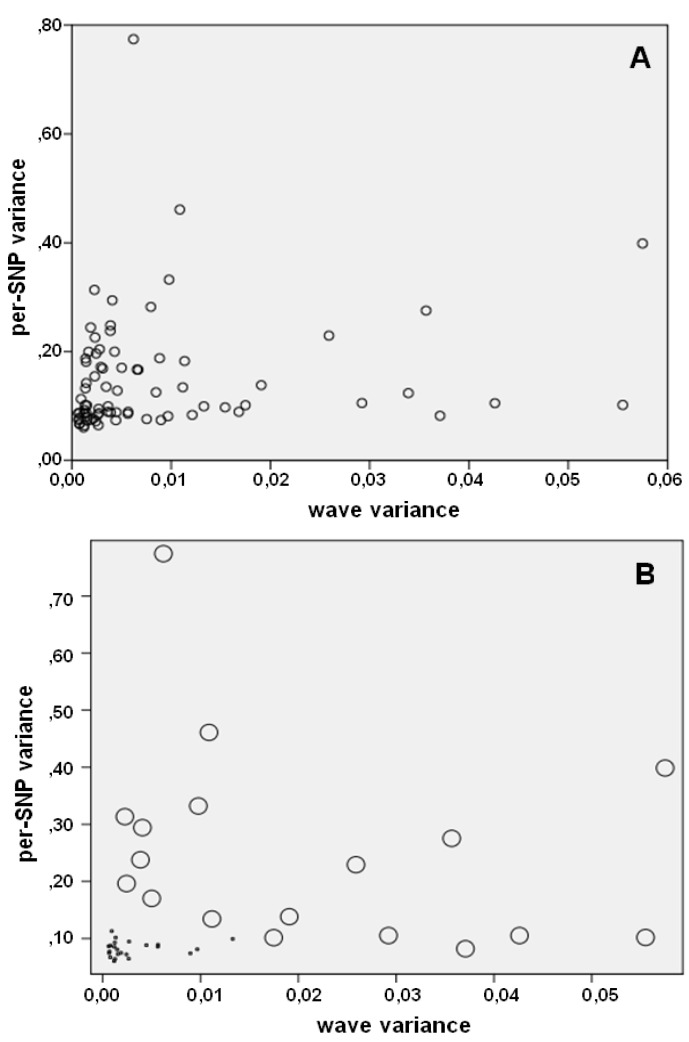
Wave variance and per-SNP variance. (**A**) Noise components in all 77 samples and (**B**) in samples of low (O) and high (●) quality (samples of intermediate quality were not included in (B)).

Figure 6 summarizes salient aspects of system noise in SNP microarrays. Figure 6(A) plots for each sample the variances of wave component and per-SNP component. Wave variance and per-SNP variance seem to occur independently from each other: the observed correlation between both noise components (*r* = 0.124) was not significant (*p* = 0.401). Figure 6(B) illustrates the relation between sample eligibility and noise components in the eligible (*n* = 23) and ineligible (*n* = 29) cases. Eligible samples (*i.e.*, those that are supposed to be excellent for copy number studies) have low levels of per-SNP variance. Samples with high wave variance are inappropriate for copy number studies. 

## 4. Noise Reduction in Copy Number Samples

**Figure 7:**
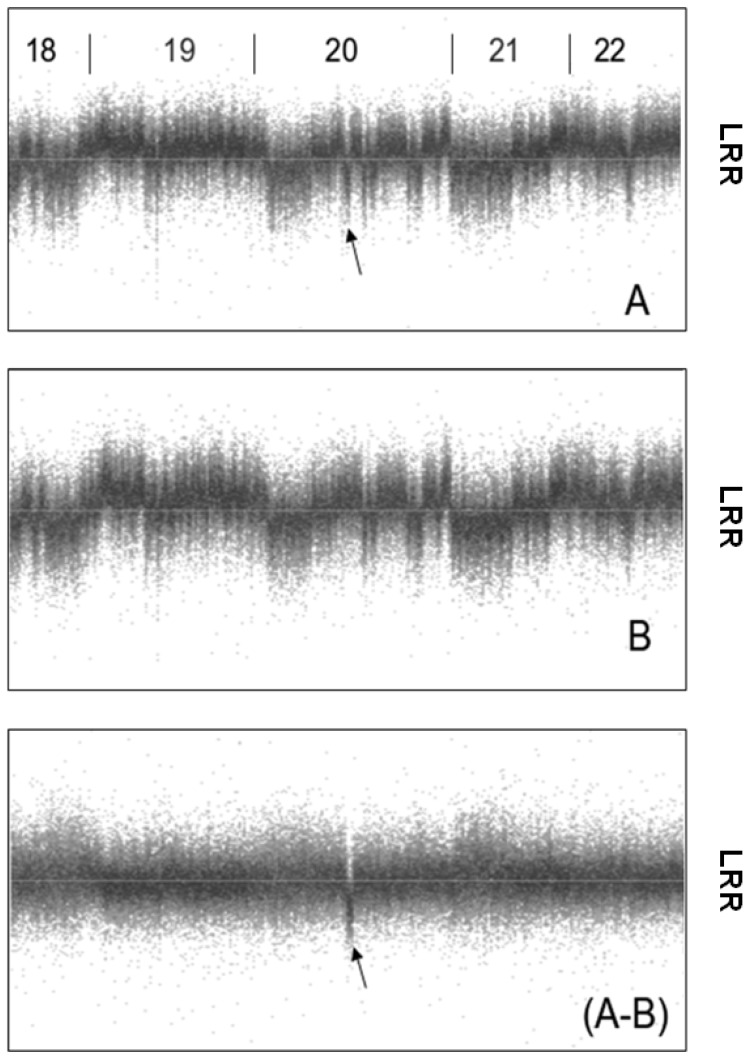
Signal intensities (y-axis: LRR values) of all SNPs from chromosome 18q up to chromosome 22. (**A**) Patient ID 1091; (**B**) reference sample ID 2355. After subtraction of the samples, a deletion in chromosome 20 became apparent (arrow).

**Figure 8:**
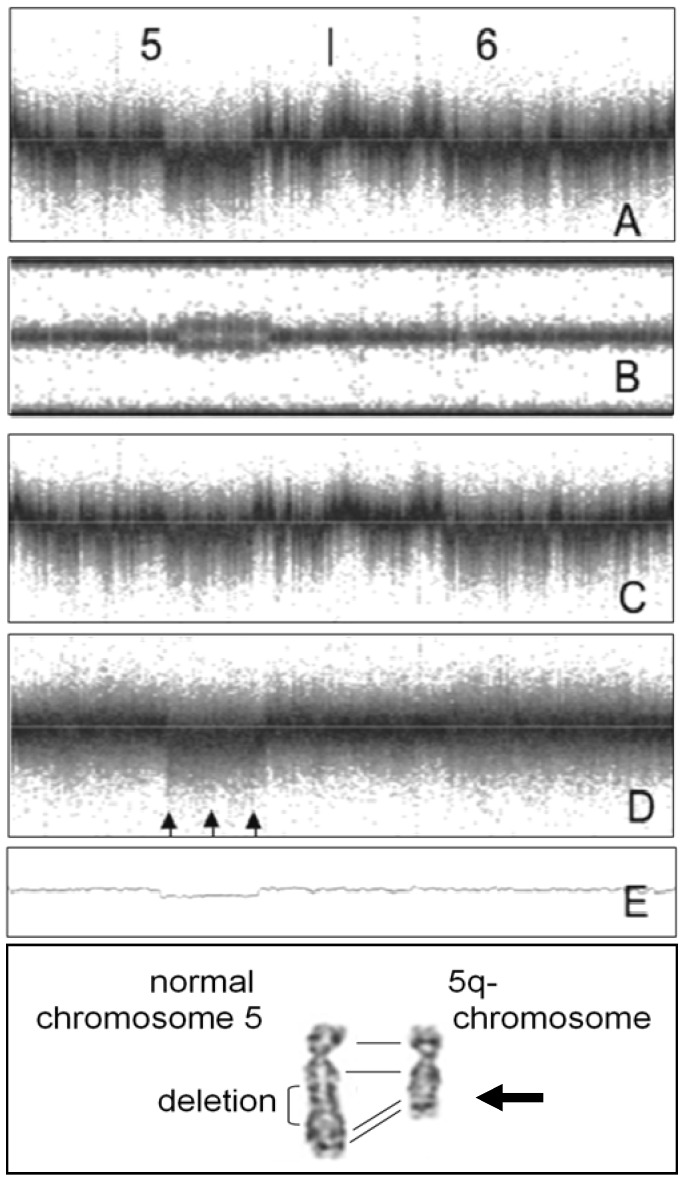
Sample with mosaic large deletion in chromosome 5q. (**A**,**B**) LRR- and BAF-values of SNPs of chromosomes 5 and 6 of patient. (**C**) LRR values of reference sample. (**D**) Signal intensities after subtraction of reference sample. Arrows indicate region with reduced LRR values. (**E**) LRR values after application of noise-free-cnv blur over 2,000 SNPs. (**Bottom panel**) Chromosome analysis of cultured peripheral blood lymphocytes from patient (courtesy of Johannes W.G. Janssen, Department of Human Genetics, University of Heidelberg). Arrow points to 5q-minus chromosome.

The noise-free-cnv software package permits the visualization of samples, the isolation of noise components and the subtraction of isolated noise components. The next two examples (Figure 7 and Figure 8) illustrate noise reduction by comparing a test sample with a reference sample. We finally demonstrate the use of the noise-free-cnv-filter algorithm for the evaluation of CNVs.

Figure 7 shows a deletion in chromosome 20 of patient ID 1091, which was detected by PennCNV and Birdview analysis. Due to strong waves, reduced signal intensities in the region of the putative deletion are not easily seen. Visual inspection of the LRR values of chromosome 20 after subtraction of a reference sample (A–B) suggested the presence of a true deletion in this patient.

Figure 8 illustrates the analysis of a mosaic deletion. Although sample ID D62 was classified as ineligible for CNV studies, analysis of SNPs with CN ≠ 2 per chromosome revealed significant clustering on chromosome 5 (Table A1; Figure 5). Neither PennCNV nor Birdsuite identified a large CNV on chromosome 5. After noise reduction, LRR and BAF values were suggestive for the presence of a mosaic deletion [22,23,24] (Figure 8(B,D)). To confirm the diagnosis of a mosaic deletion, a conventional chromosome analysis was performed: Some rare 5q chromosomes were observed amongst a majority of normal chromosome sets. Interestingly, it was recently demonstrated that the identification of mosaic abnormalities by microarray analysis is unreliable [25].

**Figure 9:**
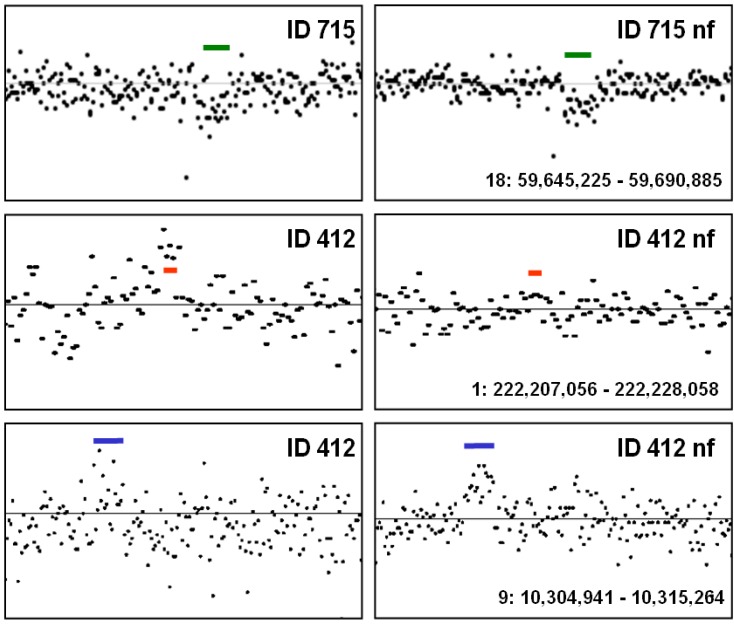
Validation of CNV findings. Left panels show crude LRR values, left panels show LRR values after noise-free-cnv-filter analysis. Samples were renamed with suffix “nf” after noise-free-cnv-filter analysis. Bars indicate putative CNV findings.

We developed the noise-free-cnv-filter algorithm for optimized noise reduction (Appendix). In the samples of our study population, noise-free-cnv-filter analysis resulted in an average reduction of the wave variance by 74.2%, of per-SNP variance by 35.3% and of the overall variance by 38.1%. Noise-reduction according to this algorithm supports the evaluation of CNV findings, in particular when the putative CNVs are small (Figure 9).

In patient ID 715, both Birdview and PennCNV identified a deletion on chromosome 18 (green bar in Figure 9). Noise-free-cnv-filter analysis of the sample (ID 715 nf) suggested that the deletion was true. Subsequent molecular analysis confirmed the finding: the joining segment of the deletion was identified by a case-specific PCR and the breakpoints of the deletion were identified by DNA sequencing following standard procedures [17,26]. Two putative duplications in patients ID 412 were evaluated after noise-free-cnv-filter analysis. We considered the duplication in chromosome 1 (region 222 Mb) as spurious (red bar), but the duplication in chromosome 9 as probably true. As a consequence, this putative duplication is a candidate for further validation by molecular methods. 

## 5. Conclusions—Proposal of a Two-Step Procedure for the Validation of CNV Findings

Our analysis had the following key findings: (1) Copy number samples may be noisy, which interferes—above a certain level of noise—with reliable identification of CNVs; (2) Eligible copy number samples were more likely when fresh DNA was used for microarray hybridization; (3) wave component and per-SNP component of noise are independent; (4) noise-free-cnv software enables noise reduction by subtracting wave and per-SNP noise components from samples; and (5) noise-free-cnv software supports the quality control of copy number data and the validation of copy number findings.

The current noise-free-cnv version was developed for the analysis of SNP microarray samples and was not designed for noise reduction in array based comparative genomic hybridization samples. The present study highlighted the value of noise reduction for large scale CNV validation (*after* software-assisted CNV detection). However, the value of noise reduction *before* software-assisted CNV detection is to be analyzed in future studies.

Based on our analysis of noise in real-life copy number samples we suggested a two-step procedure of CNV validation. As a first step of preliminary CNV validation we proposed large-scale inspection of CNV findings after noise reduction, to select putative candidate CNVs and reject false positive findings. In a second stage, this selection of putative CNV calls is analyzed further by independent molecular methods for final validation [17,26].

## Appendix: Comments to the Noise-Free-CNV Software

### A1. Noise-Free-CNV

The noise-free-cnv program package was specifically developed to analyze copy number variation in SNP-microarray samples and to manipulate the data in order to reduce noise. It was written in C++ and released as free software under the GNU General Public License version 3. Installer packages are available for Debian-based Linux systems and Windows. For the computation of the Fast Fourier Transform, we used the FFTW library [27]. Noise-free-cnv is compatible with the file format used by PennCNV [14].

The central program of the noise-free-cnv package is noise-free-cnv-gtk, a visual editor for interactive visualization and manipulation of SNP microarray data. Besides functioning as a browser for direct inspection and verification of CNV findings, it allows the user to perform many operations on the data. These include the Gaussian filters and variance computation referred to in the article. For further information, see the project homepage http://noise-free-cnv.sourceforge.net. A second program, noise-free-cnv-filter, implements a specific algorithm for system noise reduction, as described below. It is usable as a command line program to be easily applied to a batch of samples.

### A2. The Noise-Free-CNV-Filter Algorithm

The noise reduction algorithm noise-free-cnv-filter consists of two main steps. In the first step, a genomic wave profile and a per-SNP noise profile are deduced from a batch of samples. In the second step, these profiles are used to modify the individual samples.

#### A2.1. System Noise Assessment

For each individual sample:

(1) The non-autosomal data is removed and the Log R Ratio values are normalized towards an average value of zero.
(2) The wave component is computed by applying a Gaussian filter with a standard deviation of 1,000 SNPs to the Log R Ratio sequence
(3) The wave component is subtracted from the Log R Ratio values to calculate the per-SNP component.

Subsequently, the batch-specific wave is computed by regarding each SNP throughout the wave components of all samples and taking the median value. The same is done for the per-SNP profile utilizing the per-SNP components.

#### A2.2. System Noise Removal

In the second step, we use the median profiles to adjust the original samples.

For each individual:

(1) The covariance of the wave component and the batch-specific wave profile is divided by the variance of the wave profile.
(2) The result is used as a scaling factor for the wave profile, the scaled profile is then subtracted from the wave component
  The same procedure is repeated on the per-SNP components.
(3) Finally, the corrected components are added together and yield the corrected Log R Ratio values.

### A3. Program Usage

Noise-free-cnv-filter was implemented as a command-line program. In the most simple case, it receives the file names of several SNP microarray samples in the PennCNV file format (due to the nature of the algorithm, application on a single sample is pointless). It then computes the profiles (saved as “wave_profile” and “per-snp_profile”) and the cleaned versions of all provided samples, which it saves as “<original filename>.nf”. As additional options, noise-free-cnv-filter allows the use of pre-computed profile sequences and the inclusion of the sex chromosomes into the analysis. As an example, noise-free-cnv-filter—verbose individuals/* applies the algorithm to all files in the directory individuals, discards the sex chromosomes and outputs detailed information about the progress and statistical information about the samples. For further help, type: noise-free-cnv-filter—help.

**Table A1:** Eligibility of samples.

ID	call rate	var.	wave var.	per_SNP var.	Chr1	Chr2	Chr3	Chr4	Chr5	Chr6	Chr7	Chr8	Chr9	Chr10	Chr11	Chr12	Chr13	Chr14	Chr15	Chr16	Chr17	Chr18	Chr19	Chr20	Chr21	Chr22
3	98.33	0.068	0.001	0.067	2	0	0	2	2	0	0	0	0	0	0	11	0	1	0	0	2	2	0	0	0	102
15	96.02	0.144	0.001	0.142	10	4	38	8	11	2	0	50	16	11	8	150	13	0	78	22	11	21	19	0	4	0
36	96.00	0.243	0.004	0.238	506	845	678	751	1,376	503	977	974	722	385	379	639	593	232	541	752	356	397	364	225	262	203
38	95.76	0.183	0.001	0.181	179	80	102	48	140	80	91	268	33	84	122	44	27	144	20	47	114	41	155	0	40	20
48	96.16	0.174	0.007	0.167	0	39	60	141	23	42	90	69	56	5	10	59	110	18	32	361	29	32	0	94	15	55
49	94.81	0.174	0.007	0.167	203	31	35	111	40	28	33	22	14	6	22	9	8	15	41	0	0	23	12	0	7	0
50	97.14	0.103	0.002	0.101	46	11	0	14	22	242	2	0	8	0	0	65	0	0	11	2	3	0	45	0	2	2
62	97.92	0.090	0.003	0.087	0	14	0	14	3	19	15	11	2	2	0	0	0	0	0	0	6	16	0	0	0	2
71	93.92	0.202	0.002	0.200	667	490	326	78	149	40	269	252	215	116	45	231	96	163	142	266	134	148	248	38	97	50
76	93.71	0.229	0.002	0.226	511	251	200	65	229	59	336	467	352	422	457	233	85	185	170	252	285	161	462	112	167	49
97	89.52	0.291	0.008	0.282	3,123	5,467	11,613	3,795	6,729	4,870	5,374	4,898	4,455	4,169	5,721	6,492	3,486	3,020	3,709	4,031	4,120	3,177	3,581	2,466	1,775	1,281
101	97.85	0.077	0.002	0.075	0	0	0	0	0	0	0	0	0	0	0	0	0	9	0	0	0	0	0	2	0	0
111	96.51	0.175	0.003	0.172	70	76	14	49	287	187	29	76	64	266	105	47	61	17	15	20	0	74	10	136	6	21
112	94.70	0.147	0.011	0.134	2,378	2,988	2,743	2,514	2,823	4,455	2,774	3,014	2,837	2,707	4,227	3,054	2,315	1,595	1,905	1,087	2,085	2,044	1,562	650	580	602
129	96.61	0.139	0.003	0.135	7	31	51	9	19	35	3	94	30	77	23	2	3	10	0	96	0	2	74	0	22	0
131	96.45	0.199	0.002	0.196	319	229	139	314	108	125	248	304	172	363	279	68	96	93	118	212	182	71	83	28	13	28
141	96.44	0.121	0.017	0.101	266	121	174	173	147	64	195	121	258	24	43	291	45	36	59	202	105	96	208	90	86	72
144	94.72	0.176	0.005	0.170	251	746	130	464	250	328	253	410	390	139	799	55	553	63	193	178	52	286	195	9	15	45
168	94.36	0.315	0.036	0.275	5,205	5,382	5,272	4,479	4,337	5,737	6,682	4,504	3,966	2,627	2,901	5,294	2,492	3,065	2,487	3,126	2,559	2,658	2,179	1,405	961	948
182	95.02	0.316	0.002	0.313	1,189	1,988	2,051	1,096	2,301	2,991	1,814	2,365	1,408	687	1,144	1,314	839	654	689	815	569	1,953	427	517	456	377
188	90.12	0.474	0.011	0.461	14,534	15,554	28,322	10,245	10,904	16,212	12,471	14,300	6,642	6,499	8,681	9,241	5,595	6,784	7,503	5,150	6,048	7,028	3,978	2,417	3,536	2,274
189	97.32	0.097	0.012	0.084	120	34	155	12	35	41	74	69	0	29	47	34	16	6	1	0	0	0	30	0	35	2
193	97.34	0.093	0.004	0.088	53	3	0	3	0	0	0	5	71	6	2	0	47	0	0	0	0	0	0	0	0	0
412	97.51	0.092	0.006	0.086	0	0	62	2	0	0	20	0	2	14	0	10	22	0	0	0	2	2	0	0	0	4
415	98.23	0.103	0.001	0.102	5	11	0	5	3	10	35	0	2	0	0	2	0	0	0	0	0	9	0	0	0	2
421	96.73	0.165	0.055	0.102	0	7	0	0	0	5	28	0	0	0	0	0	0	2	0	0	0	4	0	0	0	0
422	96.10	0.074	0.002	0.073	37,996	36,654	32,343	29,935	29,248	29,062	23,198	24,951	21,457	22,172	22,247	21,574	15,560	14,542	14,525	12,574	10,444	14,095	8,616	10,294	7,218	4,456
430	96.39	0.160	0.019	0.138	10,614	10,096	9,196	16,278	15,383	7,959	7,758	9,465	6,757	6,295	6,291	6,559	4,955	3,487	2,491	3,459	3,074	5,404	1,525	2,399	3,569	1,187
438	89.76	0.463	0.057	0.399	45,981	56,162	42,403	45,750	55,976	37,901	44,212	34,069	30,387	26,842	26,461	38,930	23,963	20,800	18,258	19,160	16,624	18,367	9,637	13,485	8,790	5,479
442	97.82	0.084	0.008	0.076	486	382	0	0	14	0	9	57	2	0	33	4	0	0	0	0	0	0	67	0	4	3
451	95.96	0.205	0.004	0.200	109	154	49	363	75	361	107	213	124	100	85	150	124	20	63	4	60	116	50	59	0	19
461	80.86	0.706	0.007	0.696	64,822	74,302	41,655	64,993	56,987	58,825	49,369	55,433	45,357	47,372	35,753	49,377	30,870	27,158	25,448	29,590	14,637	22,231	15,372	19,289	12,431	8,971
613	97.64	0.090	0.001	0.088	2	6	3	7	4	0	49	15	0	0	22	2	10	2	0	0	0	0	7	0	2	0
647	95.49	0.157	0.002	0.154	15	1	30	166	0	47	9	0	6	45	41	58	0	5	28	41	24	0	14	4	4	11
653	98.22	0.079	0.004	0.074	12	2	14	1,618	26	7	12	7	0	9	0	0	0	0	0	2	0	0	0	0	4	4
665	97.32	0.123	0.037	0.082	5,071	8,140	6,289	5,520	6,417	6,816	5,890	6,894	4,238	5,326	4,503	5,246	2,641	2,696	2,301	1,766	1,445	4,211	1,699	1,399	2,448	932
670	96.74	0.152	0.043	0.105	3,160	4,486	3,895	3,913	3,812	3,038	3,309	3,676	2,359	2,112	3,327	2,791	1,591	1,595	1,142	969	1,028	2,043	543	1,213	1,034	286
675	97.67	0.084	0.009	0.074	0	0	3	0	4	1	3	37	0	0	58	4	0	0	0	11	0	0	48	0	0	0
676	98.15	0.078	0.001	0.077	0	0	0	0	45	2	18	0	0	0	14	4	0	0	0	0	0	0	0	0	0	0
677	95.58	0.208	0.003	0.204	25	149	161	54	77	57	267	157	38	210	63	13	52	64	34	44	57	137	121	16	56	75
693	95.72	0.133	0.005	0.128	4	73	18	6	31	12	15	27	7	0	10	8	73	14	10	0	5	3	20	2	0	12
715	97.44	0.095	0.006	0.089	3	5	0	0	4	0	471	0	2	0	0	0	0	4	0	4	0	24	0	4	0	0
717	96.60	0.134	0.001	0.132	0	2	22	21	29	41	0	17	0	6	57	10	0	2	0	15	0	0	34	0	0	0
729	94.75	0.189	0.001	0.187	115	23	115	184	49	55	84	91	42	45	101	111	16	57	103	80	57	61	192	0	22	0
733	95.84	0.198	0.009	0.188	23	28	84	218	218	0	306	180	40	34	59	11	32	3	33	62	108	6	8	38	46	0
735	95.28	0.173	0.003	0.169	94	21	111	290	81	41	116	14	220	79	0	87	47	31	34	43	16	13	181	0	0	22
742	96.83	0.114	0.013	0.099	0	0	3	0	2	0	1	0	4	0	2	0	0	15	8	2	18	0	137	0	0	0
744	97.26	0.108	0.017	0.089	0	135	126	332	73	148	263	0	135	31	52	150	180	34	15	40	0	166	114	0	94	0
746	95.72	0.253	0.004	0.248	283	895	350	378	287	388	227	723	594	712	229	559	323	206	98	478	398	689	341	73	184	173
750	97.15	0.114	0.015	0.097	2,389	642	1,750	1,501	1,370	1,792	707	1,440	997	615	674	750	559	225	544	63	187	814	458	177	184	0
752	97.80	0.103	0.004	0.099	88	121	119	155	196	83	118	187	93	206	66	147	27	35	50	41	32	70	15	36	27	25
796	94.23	0.247	0.002	0.244	2,165	482	568	1,127	539	263	360	591	440	697	908	806	106	630	256	498	262	565	127	275	67	48
1020	98.21	0.075	0.001	0.074	2	0	0	0	2	1	2	0	0	0	0	3	0	0	0	0	0	0	8	0	16	0
1022	97.77	0.082	0.001	0.081	6	16	2	30	2	2	2	0	12	0	11	0	0	0	0	0	0	1	0	0	0	0
1026	98.34	0.066	0.001	0.064	1	0	0	0	5	0	0	0	0	0	2	0	0	11	9	0	0	0	3	0	0	0
1028	97.49	0.089	0.001	0.088	9	0	0	0	40	49	0	3	0	0	3	0	0	0	0	0	0	0	0	0	0	0
1029	98.54	0.062	0.001	0.060	3	64	0	0	0	0	0	0	0	0	1	0	2	50	0	25	0	2	0	0	0	0
1033	97.58	0.087	0.001	0.085	0	0	83	0	0	0	2	0	70	4	4	0	0	0	8	0	0	0	0	0	0	0
1034	97.50	0.092	0.010	0.081	0	0	0	2	0	0	1	2	0	0	0	0	0	0	0	0	0	0	0	0	0	0
1037	98.26	0.068	0.001	0.067	8	5	2	50	2	6	2	12	0	2	1	0	2	0	0	3	54	13	2	2	0	0
1040	96.56	0.114	0.001	0.113	9	0	17	53	3	1	4	0	0	0	0	0	17	0	0	0	0	0	0	0	1	0
1041	97.16	0.094	0.001	0.093	0	0	0	4	2	2	0	0	0	0	13	3	0	0	0	0	0	0	0	0	0	0
1042	97.46	0.087	0.003	0.084	15	14	19	20	11	28	10	19	4	2	2	6	8	4	18	4	2	24	2	0	2	0
1056	97.11	0.098	0.003	0.095	2	0	2	2	0	2	0	0	0	0	0	15	0	4	0	0	35	0	10	0	0	18
1063	98.31	0.075	0.002	0.072	0	2	169	10	0	0	29	0	0	0	0	0	2	0	13	0	0	0	2	0	0	0
1065	98.23	0.068	0.003	0.065	0	4	2	7	0	0	0	2	0	0	0	0	8	0	0	0	0	58	4	0	0	0
1088	97.64	0.092	0.004	0.088	53	23	61	45	8	90	26	76	32	44	50	71	34	6	15	54	0	22	13	4	9	39
1091	96.69	0.138	0.029	0.105	7,631	6,080	7,146	6,512	7,299	4,006	4,952	6,666	4,169	2,579	3,990	4,012	2,388	2,194	1,415	2,613	1,030	2,828	646	3,697	1,862	451
1147	97.96	0.079	0.002	0.077	5	76	4	8	0	0	4	4	16	0	2	3	2	0	2	2	0	7	190	0	0	0
1151	97.90	0.087	0.001	0.086	2	4	0	0	3	0	0	8	0	4	9	0	0	0	0	0	2	0	0	0	11	2
2110	93.16	0.343	0.010	0.332	671	3,307	3,614	1,414	3,548	3,035	2,775	2,070	1,581	2,718	2,153	2,054	1,573	1,426	995	1,173	1,584	1,358	662	285	971	581
2134	95.73	0.134	0.008	0.125	144	52	81	51	51	100	28	88	70	0	35	77	3	55	7	16	56	7	41	2	18	1
2144	97.12	0.093	0.004	0.089	2	5	8	1	4	0	4	0	2	0	14	4	0	0	0	3	0	0	0	2	0	0
2240	94.48	0.299	0.004	0.294	488	1,656	1,019	1,734	2,180	1,605	754	1,750	1,179	531	916	1,728	916	751	643	921	501	427	713	356	339	374
2355	94.50	0.258	0.026	0.229	1,870	1,799	1,422	741	1,491	2,829	1,663	1,353	829	1,500	1,360	1,714	654	677	1,262	767	1,420	1,061	842	826	464	452
2406	94.78	0.195	0.011	0.183	67	125	136	122	201	80	142	109	62	61	5	51	70	68	34	7	60	83	122	53	43	0
D_062	94.17	0.322	0.003	0.318	772	838	547	536	4,419	436	711	496	239	308	219	582	319	215	222	81	62	540	101	86	0	94

For each sample, genotype call rate, variance, wave variance and per-SNP variance were calculated. The remaining columns show for each chromosome (chromosome number indicated) the number of probe sets with CN ≠ 2. This analysis included only probe sets that had normal copy number (CN = 2) in 403 samples from a population based German study (for details and references see [17]). A non-random distribution of probe sets with CN ≠ 2 is highly suggestive for the existence of a rare CNV (for instance ID 1147 or ID653, in contrast to ID 1042 or ID 2034). Even in samples with high variance, non-random distribution can be detected (chromosome 5 of ID D_062, chromosomes 1 and 2 in ID 442).

**Table A2:** Analysis of noise components in samples.

ID	variance	wave variance	per-SNP variance	wave correlation	per-SNP correlation	wave subtraction factor	per-SNP subtraction factor
3	0.068	0.001	0.067	0.804	0.676	0.409	0.800
15	0.144	0.001	0.142	0.567	0.564	0.393	0.972
36	0.243	0.004	0.238	0.877	0.388	0.997	0.865
38	0.183	0.001	0.181	0.456	0.502	0.314	0.977
48	0.174	0.007	0.167	0.939	0.508	1.405	0.949
49	0.174	0.007	0.167	0.959	0.527	1.419	0.985
50	0.103	0.002	0.101	0.881	0.536	0.626	0.779
62	0.090	0.003	0.087	0.929	0.609	0.886	0.820
71	0.202	0.002	0.200	0.365	0.589	0.274	1.203
76	0.229	0.002	0.226	0.580	0.529	0.511	1.151
97	0.291	0.008	0.282	0.875	0.360	1.427	0.873
101	0.077	0.002	0.075	0.906	0.727	0.717	0.910
111	0.175	0.003	0.172	0.914	0.482	0.903	0.914
112	0.147	0.011	0.134	0.864	0.578	1.671	0.968
129	0.139	0.003	0.135	0.898	0.620	0.964	1.043
131	0.199	0.002	0.196	0.875	0.417	0.790	0.844
141	0.121	0.017	0.101	0.949	0.703	2.297	1.024
144	0.176	0.005	0.170	0.881	0.591	1.141	1.115
168	0.315	0.036	0.275	0.936	0.406	3.234	0.975
182	0.316	0.002	0.313	0.809	0.386	0.704	0.990
189	0.097	0.012	0.084	0.930	0.668	1.874	0.883
193	0.093	0.004	0.088	0.960	0.704	1.174	0.956
412	0.092	0.006	0.086	0.950	0.691	1.304	0.925
421	0.103	0.001	0.102	0.898	0.680	0.598	0.991
422	0.165	0.055	0.102	0.873	0.523	3.763	0.763
425	0.074	0.002	0.073	0.908	0.572	0.653	0.705
430	0.160	0.019	0.138	0.896	0.457	2.265	0.778
438	0.463	0.057	0.399	0.913	0.354	4.006	1.021
442	0.084	0.008	0.076	0.942	0.663	1.496	0.835
451	0.205	0.004	0.200	0.927	0.453	1.111	0.927
461	0.706	0.007	0.696	−0.310	0.228	−0.460	0.868
613	0.090	0.001	0.088	0.850	0.565	0.589	0.767
647	0.157	0.002	0.154	0.766	0.589	0.671	1.059
653	0.079	0.004	0.074	0.938	0.569	1.141	0.707
665	0.123	0.037	0.082	0.896	0.555	3.159	0.726
670	0.152	0.043	0.105	0.906	0.565	3.422	0.837
675	0.084	0.009	0.074	0.951	0.672	1.647	0.837
676	0.078	0.001	0.077	0.646	0.635	0.313	0.807
677	0.208	0.003	0.204	0.901	0.465	0.870	0.960
693	0.133	0.005	0.128	0.953	0.581	1.179	0.952
715	0.095	0.006	0.089	0.950	0.667	1.308	0.911
717	0.134	0.001	0.132	0.522	0.650	0.351	1.081
729	0.189	0.001	0.187	0.606	0.611	0.411	1.209
733	0.198	0.009	0.188	0.947	0.488	1.628	0.967
735	0.173	0.003	0.169	0.901	0.609	0.917	1.144
742	0.114	0.013	0.099	0.956	0.649	2.017	0.936
744	0.108	0.017	0.089	0.921	0.570	2.186	0.779
746	0.253	0.004	0.248	0.906	0.414	1.033	0.943
750	0.114	0.015	0.097	0.940	0.612	2.137	0.874
752	0.103	0.004	0.099	0.937	0.471	1.033	0.679
796	0.247	0.002	0.244	0.015	0.527	0.012	1.192
1020	0.075	0.001	0.074	0.742	0.614	0.348	0.767
1022	0.082	0.001	0.081	0.909	0.644	0.640	0.836
1026	0.066	0.001	0.064	0.861	0.664	0.557	0.770
1028	0.089	0.001	0.088	0.742	0.643	0.380	0.871
1029	0.062	0.001	0.060	0.912	0.661	0.572	0.742
1033	0.087	0.001	0.085	0.820	0.703	0.518	0.940
1034	0.092	0.010	0.081	0.963	0.709	1.732	0.924
1037	0.068	0.001	0.067	0.782	0.633	0.390	0.748
1040	0.114	0.001	0.113	0.639	0.701	0.355	1.077
1041	0.094	0.001	0.093	0.850	0.672	0.546	0.937
1042	0.087	0.003	0.084	0.947	0.695	0.884	0.921
1056	0.098	0.003	0.095	0.941	0.686	0.893	0.966
1063	0.075	0.002	0.072	0.924	0.571	0.832	0.701
1065	0.068	0.003	0.065	0.959	0.657	0.904	0.764
1088	0.092	0.004	0.088	0.918	0.497	1.046	0.675
1091	0.138	0.029	0.105	0.912	0.537	2.854	0.797
1147	0.079	0.002	0.077	0.944	0.717	0.795	0.909
1151	0.087	0.001	0.086	0.688	0.572	0.311	0.769
2110	0.343	0.010	0.332	0.948	0.408	1.715	1.075
2134	0.134	0.008	0.125	0.936	0.551	1.575	0.893
2144	0.093	0.004	0.089	0.953	0.609	1.046	0.832
2240	0.299	0.004	0.294	0.909	0.433	1.060	1.075
2355	0.258	0.026	0.229	0.960	0.530	2.826	1.161
2406	0.195	0.011	0.183	0.940	0.570	1.833	1.113
188c	0.474	0.011	0.461	0.899	0.314	1.715	0.975
D62	0.322	0.003	0.318	0.819	0.340	0.761	0.878
